# A robust ammonia metabolism gene signature identified by machine learning predicts prognosis and immunotherapy response in clear cell renal cell carcinoma

**DOI:** 10.3389/fonc.2025.1709096

**Published:** 2026-01-08

**Authors:** Zhilin Gong, Hansen Lin, Jue Wang, Jintao Hua, Jinhuan Wei, Wei Chen, Junhang Luo, Jun Pang, Xu Chen

**Affiliations:** 1 Department of Urology, The First Affiliated Hospital of Sun Yat-Sen University, Guangzhou, Guangdong, China; 2 Department of Urology, Kidney and Urology Center, Pelvic Floor Disorders Center, The Seventh Affiliated Hospital, Sun Yat-Sen University, Shenzhen, Guangdong, China; 3 Department of Pathology, The First Affiliated Hospital of Sun Yat-sen University, Guangzhou, Guangdong, China

**Keywords:** ammonia-metabolism, clear cell renal cell carcinoma (ccRCC), tumor immune microenvironment, immunotherapy, prognostic prediction model

## Abstract

**Background:**

Despite the widespread use of immune checkpoint inhibitors (ICIs) in advanced clear cell renal cell carcinoma (ccRCC), therapeutic resistance persists. The prognostic and immunomodulatory role of ammonia metabolism remains unclear.

**Methods:**

We leveraged public RNA-seq data and machine learning to identify ammonia metabolism pathways through enrichment analysis of programmed cell death-related genes. Employing multi-omics data from ccRCC patients, we developed an ammonia metabolism risk score (AMRS) via machine learning, which was validated externally and in immunotherapy cohorts. Additionally, scRNA-seq, WGCNA, TMB analysis, and *in vitro* assays were performed to characterize the model’s functional basis.

**Results:**

From 147 prognostic ammonia metabolism-related genes in TCGA, a 4-gene random forest model was constructed using LASSO and multivariate Cox regression. This model demonstrated robust predictive accuracy in external validation (3/5/7-year AUCs: 0.710/0.721/0.771). High-risk patients showed significantly elevated mortality in external cohorts (HR = 4.23, 95% CI 1.57–11.42, p = 0.002) and multiple ICI cohorts (HR = 1.30–1.69, p < 0.05). Functional validation via CSAD-targeted siRNA knockdown suppressed migration and invasion by >44% (p < 0.05) across four ccRCC cell lines.

**Conclusions:**

Our integrated approach overcomes modeling constraints from limited samples and high-dimensional data and establishes a novel ammonia metabolism-related prognostic signature for ccRCC. CSAD emerges as a promising biomarker warranting further investigation.

## Introduction

Clear cell renal cell carcinoma (ccRCC) is the most prevalent renal malignancies, characterized by rising incidence and mortality (1, 2). The poor prognosis of metastatic ccRCC, is closely linked to immune escape driven by altered immune microenvironments. This phenotype can be mediated through diverse cell death pathways. Although Immune checkpoint inhibitors (ICIs) represent a primary treatment approach, their efficacy is frequently limited by therapy resistance mechanisms (3, 4).

It has been demonstrated that aberrant ammonia metabolism contributes to tumor immune escape, with elevated ammonia concentrations being a significant cause of effector CD8+ T cell death (5). In the TME, ammonia production is primarily derived from glutamine catabolism. ccRCC tumor cells undergo glutamine metabolic reprogramming, which not only mitigates ammonia toxicity but also provides a nitrogen source for tumor cell proliferation (6–8). Simultaneously, the substantial uptake of glutamine by tumor cells leads to local glutamine depletion, which can stimulate regulatory T cell proliferation and thereby suppress anti-tumor immune responses (9).

The International Metastatic Renal Cell Carcinoma Database Consortium (IMDC) model, primarily based on clinical parameters without incorporating molecular features, demonstrates limited predictive capacity for ICI therapy response (10). Machine learning approaches represent a promising strategy for developing novel prognostic models that integrate molecular features. Machine learning has demonstrated promising efficacy in malignancies that possess larger samples. This is evidenced by a lung adenocarcinoma (LUAD) prognostic study in which the CoxBoost+plsRcox methodology was applied, that six validation cohorts achieved mean 1/3/5-year AUC of 0.76/0.73/0.73 (11). Similarly, in hepatocellular carcinoma (HCC), a predictive model constructed via StepCox+GBM attained 1/3-year AUC of 0.74/0.78 in a validation cohort (12). However, developing robust models for low-prevalence malignancies such as ccRCC, which suffers from limited public datasets remains challenging. For instance, a study on prognostic analysis of ccRCC employing random forest methodology yielded mean 1/3/5-year AUC of 0.73/0.694/0.672 across three validation cohorts (13). Significant AUC disparities at the 1-year and 3-year between ccRCC and high-incidence cancers (LUAD/HCC) were observed, necessitating methodological refinements.

Screening biomarkers derived from cell death and tumor immunotherapy mechanisms represents a promising strategy. This approach is exemplified by a breast cancer study in which programmed cell death (PCD) genes were used to construct a predictive model demonstrating efficacy for prognosis and drug sensitivity assessment (14).

In ccRCC, most existing studies have concentrated on a single form of cell death, such as ferroptosis or disulfidptosis, and developed prognostic models based on genes associated with these pathways, thereby providing valuable insights into disease progression (15, 16). In contrast, we seek to adopt a more systemic approach for exploring the potential roles of programmed cell death and ammonia metabolism in the regulation of the tumor microenvironment. Accordingly, the final model developed in this work incorporates genes involved in multiple stages of ammonia metabolism—including generation, transport, and detoxification—as well as immunomodulatory genes that may interact with ammonia metabolism. This multi−aspect integration strategy is intended to more comprehensively elucidate the complex functions of ammonia metabolism in immune regulation. This multi-aspect integration strategy, in contrast to focusing on a single death pathway such as ferroptosis or disulfidptosis, may allow a more comprehensive elucidation of the complex functions of ammonia metabolism in immune regulation and could help uncover alternative mechanisms underlying the formation of an immunosuppressive tumor microenvironment.

Employing a cell death gene screening strategy, this study developed a novel prognostic model designed to predict ccRCC outcomes and ICI therapeutic response through integration of traditional LASSO/Cox regression with machine learning methodologies based on ammonia metabolism-related genes (AMGs), subsequently validated in external cohorts. This integrated strategy represents a potential way for predictive modeling in malignancies characterized by limited samples but high-dimensional transcriptomic profiles, such as ccRCC. Concurrently, biomarker exploration was conducted to identify potential therapeutic targets.

## Materials and methods

### Data collection

The gene expression profiles and clinical data of 614 ccRCC patients were downloaded from the cancer genome atlas (TCGA) database (72 normal tissues; 542 tumor tissues, https://cancergenome.nih.gov/). After 7 patients with incomplete survival data were excluded, 535 tumor samples were used for further analysis. The single-cell RNA sequencing (scRNA-seq) data of tumor tissues from 7 ccRCC patients were downloaded from the gene expression omnibus (GEO) database (GSE159115, https://www.ncbi.nlm.nih.gov/geo/). R software was used for data integration and normalization.

E-MTAB-1980 from ArrayExpress (https://www.ebi.ac.uk/arrayexpress/) was utilized for external validation of the model.

Three ICI treatment cohorts were evaluated: the CheckMate-025 cohort (17), which included standardized transcriptomic and clinical data from RCC patients treated with everolimus or nivolumab; the IMmotion150 cohort (18), which included standardized transcriptomic and clinical data from RCC patients treated with atezolizumab; the IMmotion151 cohort (19, 20), which included standardized transcriptomic and clinical data from RCC patients treated with atezolizumab plus bevacizumab.

1,471 PCD-related genes and 620 AMGs were collected from the literature (5, 14, 21–23), and molecular signatures database (MSigDB) (v2023.2; http://www.gsea-msigdb.org)(see **Supplementary Tables S1**, **S2**) (24).

### Construction of a preliminary model

Within the TCGA cohort, significant DEGs between the tumor and normal groups were identified using the “DESeq2” R package, with |log2FC| ≥ 1 and a false discovery rate (FDR) < 0.05 (25). These DEGs were intersected with PCD-related genes, followed by survival screening via the “survival” R package, and subsequently through six machine learning methodologies for preliminary modeling.

### Validation of the preliminary model

Predictive performance of the preliminary model was validated using the C-index, Kaplan-Meier curves, and time-dependent ROC curves, implemented through the “survival”, “timeROC”, and “ggplot2” R packages. To explore factors that influence model performance, Kyoto encyclopedia of genes and genomes (KEGG)/gene ontology (GO) enrichment analysis was performed via “clusterProfiler” on genes incorporated into the machine learning, with subsequent visualization using “ggplot2”.

### Weighted gene coexpression network analysis

Following preprocessing of TCGA RNA-seq data with outlier removal, a correlation matrix was constructed using the “WGCNA” R package, where the optimal soft thresholding power was determined to establish a scale-free network. This threshold converted the matrix into an adjacency form, from which a topological overlap matrix (TOM) was derived. Using TOM-based dissimilarity and average linkage hierarchical clustering, genes were categorized into coexpression modules. Functional enrichment analysis was performed on the module which exhibited the highest correlation, to explore associated biological mechanisms.

### Analysis of scRNA-seq data

Single-cell expression matrices were constructed using the “Seurat” R package, retaining cells that passed quality control thresholds (genes: 200–5,000; unique molecular identifier: 200–30,000; mitochondrial genes <20%; hemoglobin genes <5%). Log-normalization was applied with a 10,000 scale factor, identifying the top 2,000 highly variable genes for downstream analysis. Principal component analysis (PCA) was performed on these genes, with batch effect correction using the “harmony” package, followed by application of the t-distributed stochastic neighbor embedding (t-SNE) method for dimensionality reduction. Cluster-specific marker genes were identified via “FindAllMarkers”, enabling manual cell-type annotation using the CellMarker database and literature (26, 27). CD8^+^ T cells were isolated based on annotations, and DEGs between CD8^+^ T cells and other populations (|log_2_FC|≥1, FDR <0.05) were identified using “FindAllMarkers”. GO/KEGG enrichment for DEGs was conducted via “clusterProfiler” with”ggplot2” visualization.

### Constructing the ammonia metabolism risk score

Employing a methodology analogous to PCD-related gene screening, AMGs were filtered for further analysis. Subsequently, LASSO regression analysis was performed using the “glmnet” R package (28), which was combined with traditional multivariate Cox regression and machine learning (random forest) methodologies to construct the AMRS prognostic model.

### Validation of the AMRS

Predictive performance of the AMRS was evaluated in an external validation cohort (E-MTAB-1980), where time-dependent ROC and Kaplan-Meier survival curves were employed for validation. To enable subsequent performance comparisons, the traditional multivariate Cox regression model was evaluated in the same cohort.

### Copy number variation and tumor mutational burden analysis

Using the CNV profiles and somatic mutation data from ccRCC patients in the TCGA database, the “maftools” R package was employed to conduct mutation analysis and detect CNV alterations in model construction genes, which were visualized using a waterfall plot (29). The chromosomal distribution patterns of these genes were mapped using the “RCircos” R package to generate Circos plots. TMB scores were subsequently calculated for each patient, and Spearman’s correlation analysis was used to assess the relationship between TMB scores and AMRS.

### Clinical correlation and drug sensitivity analysis

The distributions of the clinical features across different risk groups were analyzed, and the results are presented in bar plots.

In order to estimate drug sensitivity across different risk groups, the “oncoPredict” R package was used along with the drug sensitivity data from the GDSC to calculate the drug sensitivity scores in each risk group, with the top 9 most significant results visualized in box plots (30).

### AMRS and response to ICI treatment

In order to further investigate the relationship between AMRS and the tumor immune microenvironment (TIME) in ccRCC, as well as its implications for ICI therapeutic efficacy, the Tumor immune dysfunction and exclusion (TIDE), ESTIMATE, and CIBERSORT algorithms were employed to profile TIME characteristics across distinct risk strata. Simultaneously, the established AMRS was applied to three independent ICI treatment cohorts to evaluate risk stratification utility via survival analysis.

### Cell culture and transfection

Human ccRCC cell lines (786-O, 769-P, A498, and OSRC2) were obtained from the American type culture collection (ATCC) and cultured in RPMI-1640 medium supplemented with 10% fetal bovine serum (FBS) at 37°C with 5% CO_2_.

The small interfering RNA negative control (si-NC), cysteine sulfinic acid decarboxylase CSAD-targeting siRNA1 (siCSAD-1) and CSAD-targeting siRNA2 (siCSAD-2) were transfected into cells using the jetPRIME reagent, following the protocol provided by the manufacturer.

### Real-time reverse transcription quantitative polymerase chain reaction

Total RNA extracted from transfected cells or tumor tissues and paired adjacent normal samples was reverse-transcribed to synthesize cDNA, which served as the template for RT-qPCR analyzedvia the 2^(−ΔΔCT) method. This study utilized commercial kits, including RNA extraction: EZ-press RNA Purification Kit (No.B0004D-50, EZBioscience); cDNA synthesis: RT kits with gDNA remover (No.A0010GQ, EZBioscience); RT-qPCR amplification: 2×SYBR Green RT-qPCR Master Mix kit (No.A0010GQ, EZBioscience). The primers used in this study were as follows: CSAD: 5’-TTTGGGGCTTGCTCCTACCT-3’ (forward) and 5’-GCTCCTTCCACTCACAGACC-3’ (reverse). IL4I1: 5’-CGTGCAGATCGAGACCTCTC-3’ (forward) and 5’-CCTTTTCGGTTTGCCAGAGC-3’ (reverse). PSAT1: 5’-CAGTTCAGTGCTGTCCCCTT-3’ (forward) and 5’-GCGGCACCTCCATTGTTTTT-3’ (reverse). P4HA3: 5’-GAGCAAGACCTTCCAGCCTT-3’ (forward) and 5’-AGGTGGGGTATATTGGGCCT-3’ (reverse). ACTB was used as the reference gene, and the primer sequences were: 5’-CATGTACGTTGCTATCCAGGC-3’ (forward) and 5’-CTCCTTAATGTCACGCACGAT-3’ (reverse).

The study protocol involving human specimens was approved by the Institutional Ethics Committee for Clinical Research and Animal Trials of the First Affiliated Hospital of Sun Yat-sen University (Approval No. (2022)681) and conducted in accordance with the ethical principles of the Declaration of Helsinki.

### Western blot

Total protein was extracted using RIPA lysis buffer(Beyotime)supplemented with protease and phosphatase inhibitors (MCE), with incubation on ice for 15 minutes. The lysates were then centrifuged at 12,000 × g for 2 minutes at 4°C, and the supernatant was collected for protein concentration determination using a BCA protein assay kit (ThermoFisher, USA). Proteins were denatured with 5 × SDS-PAGE loading buffer, separated by electrophoresis on 12% SDS-polyacrylamide gels, and subsequently transferred onto 0.2 μm PVDF membranes (Millipore). The membranes were blocked with skim milk for 1 h, followed by incubation with primary antibodies at 4°C for 12 h and then with corresponding secondary antibodies at room temperature for 1 h. The antibodies used in this study were as follows: CSAD (1:1000, K113270P, Solarbio|ActivAb).

### Colony formation assay

siRNA-transfected 786-O, 769-P, A498, and OSRC2 cells were cultured in RPMI-1640 medium supplemented with 10% FBS at 37 °C under 5% CO_2_. At 24 hours post-transfection, 1000 cells from each cell line were seeded per well of 6-well plates and maintained in RPMI-1640 medium containing 10% FBS for two weeks. Colonies were subsequently counted and analyzed.

### Wound healing assay

When 786-O, 769-P, A498, and OSRC2 cells reached approximately 90% confluence in 6-well plates, a sterile 200 μL pipette tip was used to create a linear wound scratch across the monolayer. The cells were then gently washed with PBS to remove detached cells and replenished with serum-free medium. Wound images were captured at 0 h and 12 h after scratching using an inverted microscope. The relative wound closure rate was quantified using ImageJ software.

### Transwell migration and invasion assay

Uncoated or Matrigel-coated transwell chambers (Corning, USA) were placed into 24-well plates to assess cell migration or invasion. A total of 100 µL of serum-free medium containing re-suspended cells was added to the upper chamber. Meanwhile, 700 µl of PRMI 1640 medium containing 10% FBS was added into the lower chamber. Following this, the cells for migration assays were incubated for 8h, and cells for invasion assays were incubated for 6h, those migrated/invaded cells were stained with 0.4% crystal violet solution before imaging for quantification.

### Statistical analysis

Statistical analyses were conducted, and the results were visualized using R software (version 4.4.1). A p-value of < 0.05 was considered statistically significant.

## Results

### Analysis based on preliminary model

Initially, this study attempted to develop a prognostic model based on PCD-related genes to predict ccRCC patients’ response to ICI.

**Figure 1:**
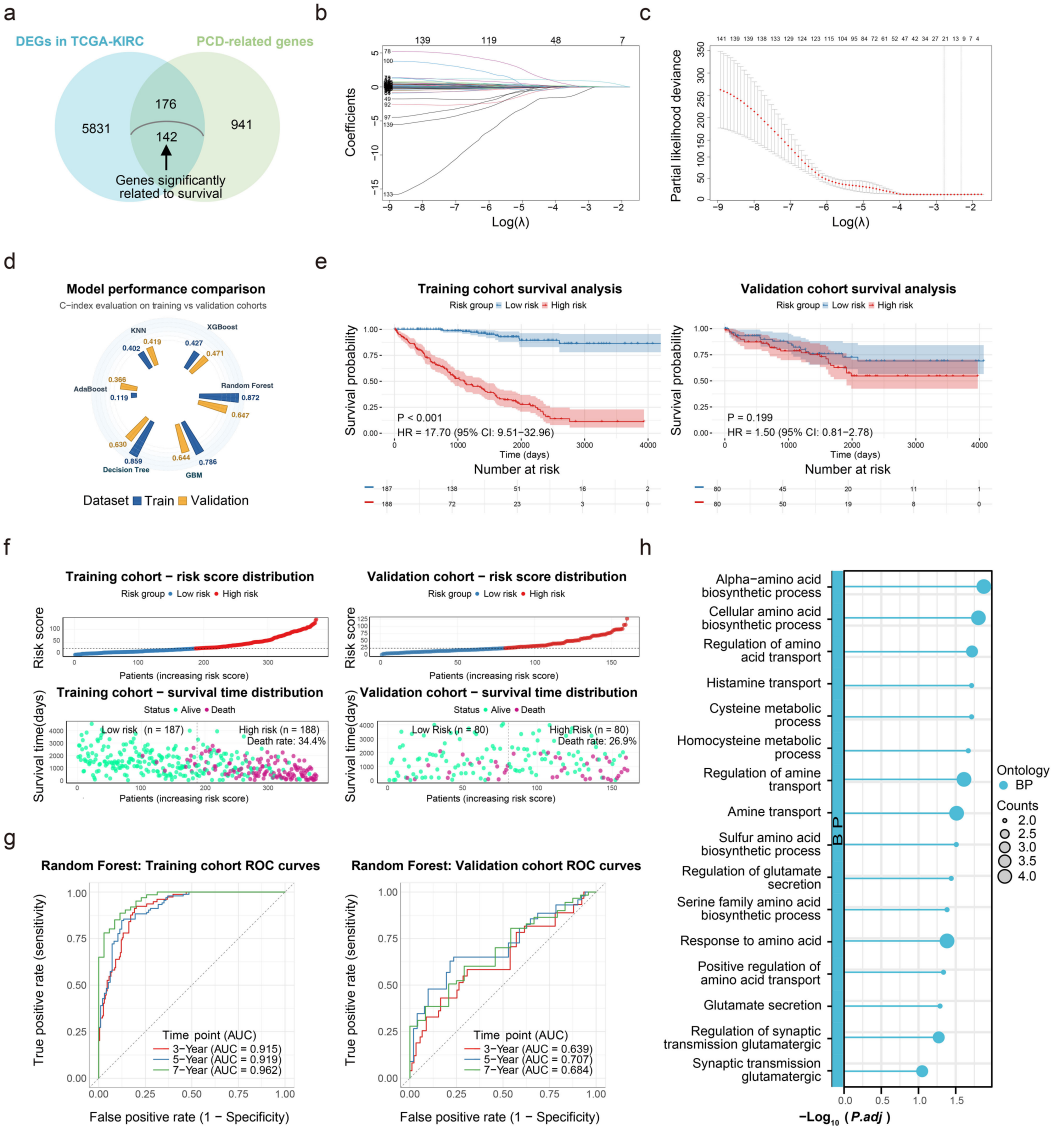
Use machine learning to construct a prognostic prediction model for ccRCC using PCD-related genes, **(a)** Perform initial screening for PCD-related genes significantly associated with the prognosis of ccRCC. **(b)** Machine learning model construction by LASSO regression analysis. **(c)** Cross-validation for the minimum lambda value in the LASSO regression model. **(d)** Performance comparison of 6 machine learning models. **(e)** Survival analysis using the random forest model in the training and validation cohorts. **(f)** Survival distribution in the training and validation cohorts. **(g)** 3/5/7-year ROC curves for the random forest model. **(h)** KEGG/GO enrichment analysis of 142 PCD prognostic genes. .

Significant DEGs were identified between tumor and normal tissues (2,017 downregulated; 4,132 upregulated), which were intersected with 1,259 known PCD-related genes, producing 318 overlapping genes, among which 142 were significantly associated with OS (**Figure 1a**). Subsequent LASSO regression analysis was performed, and the results were incorporated into six machine learning methods to construct preliminary models (**Figures 1b–d**). The random forest model was selected for its highest C-index (0.647) in internal validation, with patients stratified into high/low-risk groups based on median risk scores to further validate model performance (**Figure 1d**). As depicted in **Figures 1e-g**, the random forest model demonstrated strong predictive performance in the training cohort, while its performance in the internal validation cohort was suboptimal. In the internal validation cohort, the Kaplan-Meier curve failed to reach statistical significance (p > 0.05) for stratifying patient OS, with relatively low AUC values further evidencing limited predictive efficacy.

To investigate this performance discrepancy, KEGG/GO enrichment analysis was conducted on the 142 PCD-related genes, which were associated with OS, revealing significant enrichment in amino acid and ammonia metabolism pathways (**Figure 1h**). Prompted by this association, the study focus shifted to explore AMGs roles in ccRCC prognosis and ICI therapeutic efficacy.

### WGCNA and scRNA-seq analysis

**Figure 2:**
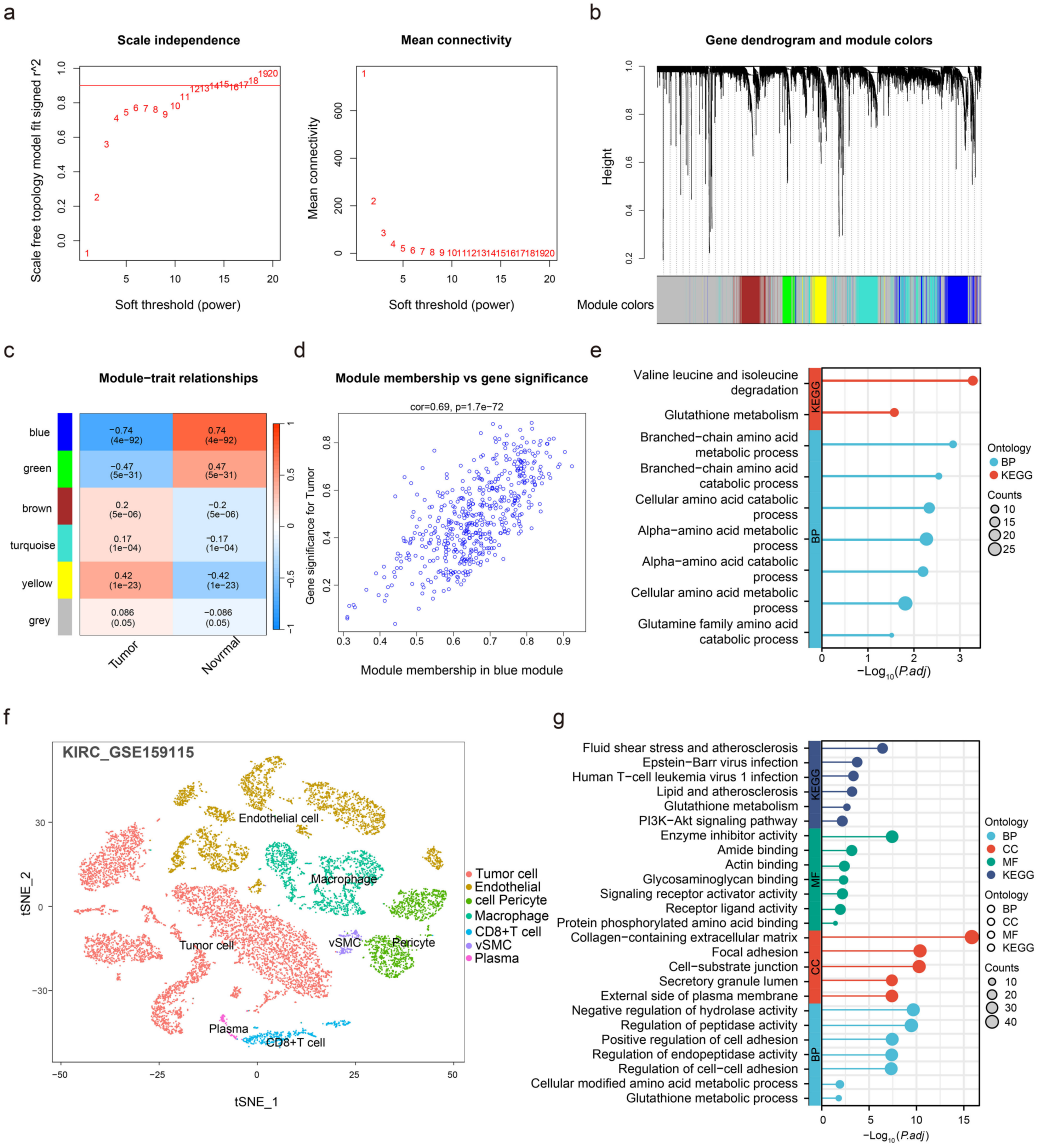
Utilize weighted gene co-expression network analysis (WGCNA) to identify highly correlated gene modules, and further validate the association of AMGs with ccRCC using scRNA-seq data analysis. **(a)** Soft thresholding power determination for constructing a scale-free network, showing the scale-free topology fit index and mean connectivity for different power values. **(b)** Cluster dendrogram of the top 30% most variable genes in the TCGA-KIRC grouped into distinct modules, with colors representing module membership. **(c)** Module-trait relationships illustrating correlations between modules (e.g., blue, green, brown) and clinical traits, such as tumor presence. **(d)** Scatter plot showing the correlation between module membership and gene significance in the blue module, which is highly associated with ccRCC. **(e)** KEGG/GO pathway enrichment analysis for genes in the blue module. **(f)** Annotation and clustering of all cell types in GSE159115. **(g)** The differential gene KEGG and GO enrichment analysis results of CD8+ T cells compared to other cell types. .

The soft-threshold power of 12 was calibrated, followed by identification of six modules via WGCNA (**Figures 2a-c**), among which the blue module demonstrated the strongest tumor correlation (**Figures 2c, d**). Consequently, genes from this module were subjected to KEGG/GO enrichment analysis, revealing significant enrichment in glutathione metabolism and cellular amino acid catabolic processes, thereby demonstrating AMGs involvement in ccRCC tumorigenesis (**Figure 2e**).

To delineate cellular-level associations between AMGs and ccRCC, scRNA-seq data from seven ccRCC patients (GSE159115) were analyzed. Dimensionality reduction and clustering identified seven distinct cell populations in ccRCC tissues (**Figure 2f**). Differential gene expression analysis comparing CD8^+^ T cells with other cell types revealed 397 DEGs, which on KEGG/GO enrichment analysis exhibited significant enrichment in ammonia metabolism processes (**Figure 2g**), indicating close associations between CD8^+^ T cells and ammonia metabolism at single-cell resolution.

### Construction of the AMRS prognostic model

**Figure 3:**
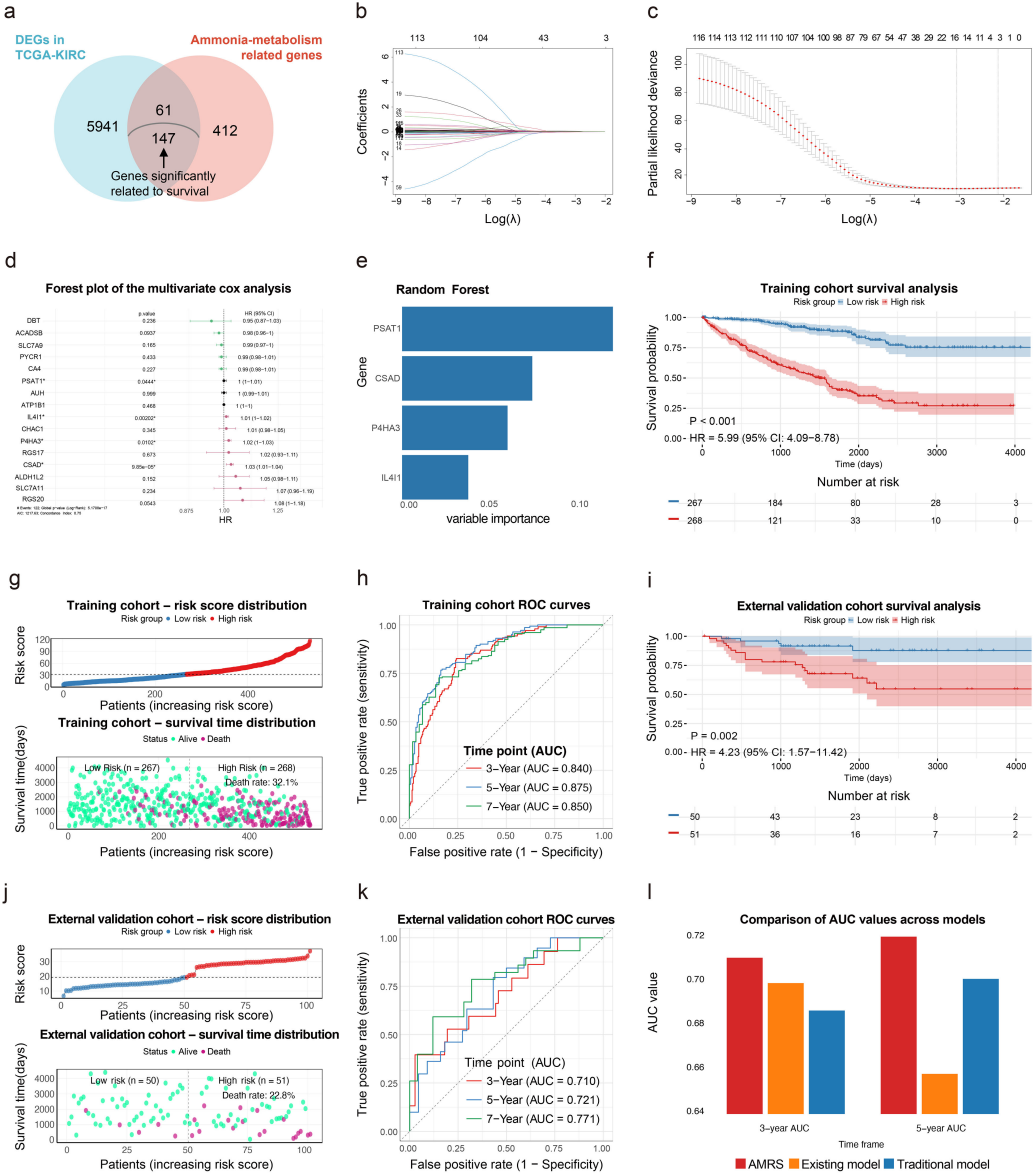
Use Random Forest model to construct a prognostic prediction model for ccRCC using AMGs. **(a)** Venn diagram of the selection process for AMGs. **(b)** LASSO regression analysis. **(c)** Cross-validation for the minimum lambda value in the LASSO regression analysis. **(d)** Forest plot presenting multivariate Cox regression analyses following LASSO regression. **(e)** The importance of Random Forest variables. **(f)** Survival analysis in the training cohort. **(g)** Survival distribution in the training cohort. **(h)** 3-/5-/7-year ROC curves for the training cohort. **(i)** Survival analysis in the external validation cohort (E-MTAB-1980 cohort). **(j)** Survival distribution in the external validation cohort (E-MTAB-1980 cohort). **(k)** 3-/5-/7-year ROC curves for the external validation cohort (E-MTAB-1980 cohort). **(l)** Comparison of AUC values across models.

Ultimately, 147 AMGs, which were associated with OS, were identified for subsequent analysis (**Figure 3a**). LASSO regression followed by multivariate Cox regression was performed on these genes (**Figures 3b-c**), yielding four statistically significant genes were integrated into a random forest model to construct the AMRS (**Figure 3d**), with variable importance measures presented in **Figure 3e**.

### AMRS validation and multi-model performance comparison

Survival analysis revealed significantly worse prognoses in high-risk versus low-risk patients (p<0.05, **Figures 3f, g, i, j**). Additionally, ROC curves demonstrated robust AMRS predictive performance, with all AUC values exceeding 0.7 and some surpassing 0.8 (**Figures 3h, k**).

To further compare predictive performance among AMRS, the traditional model (see **Supplementary Figure S1**), and the existing ammonia-related gene model (13), all models were validated in the identical E-MTAB-1980 cohort, with comparative results shown in **Figure 3l**. AMRS demonstrated superior predictive efficacy at 3- and 5-year timepoints among the three models.

### CNV and TMB analysis based on the AMRS

**Figure 4:**
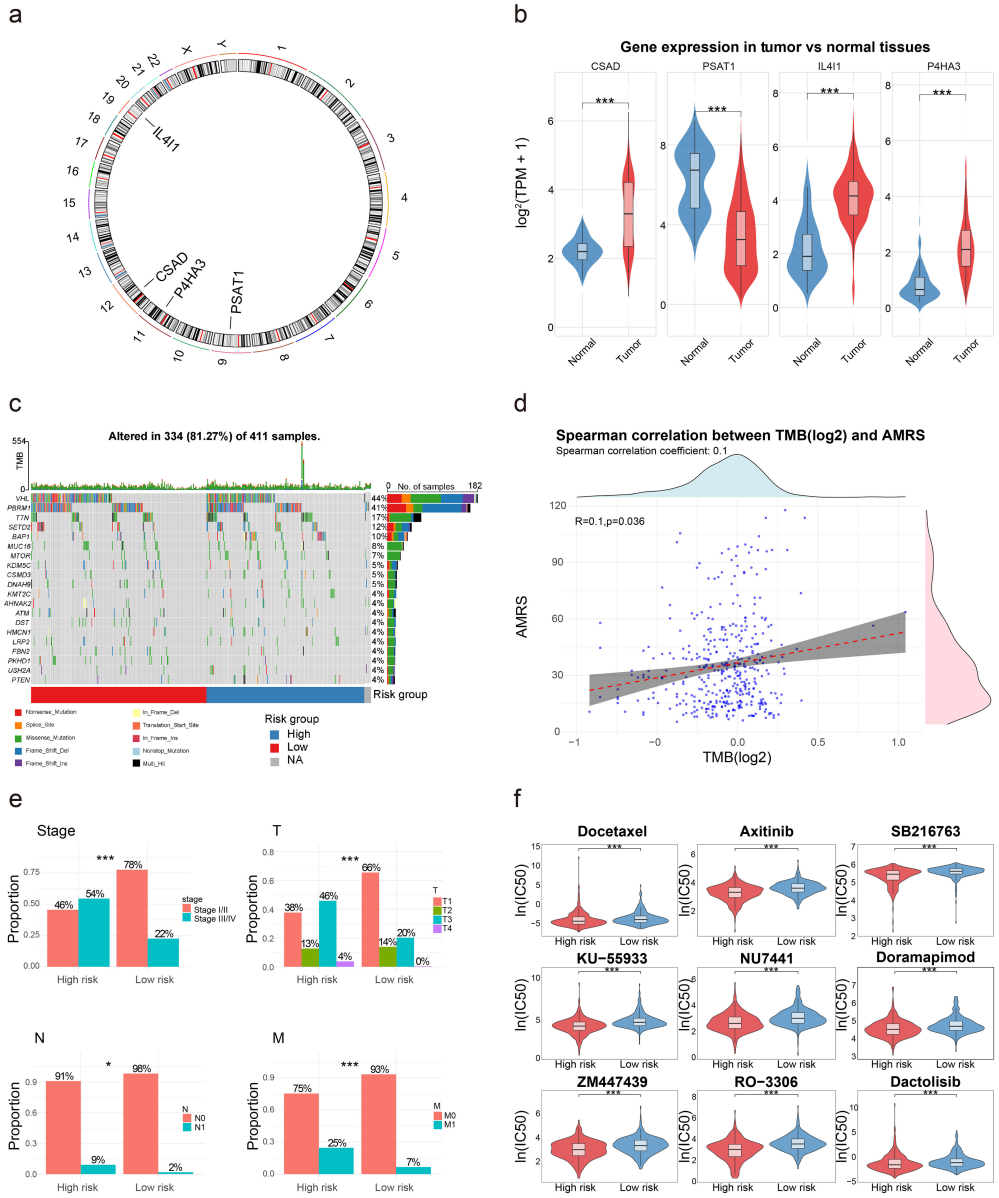
Multi-omics landscape of the AMRS genes in ccRCC. **(a)** The circus plot of CNV on chromosome location in 4 AMRS genes. **(b)** Expression profiles of 4 AMRS genes between ccRCC tissues and normal tissues in the TCGA-KIRC cohort. **(c)** Waterfall plots of mutation characteristics based on AMRS. **(d)** Spearman correlation analysis between AMRS and TMB. **(e)** Clinical correlation analysis in TCGA-KIRC cohort based on AMRS. **(f)** Drug sensitivity analysis based on AMRS. *P < 0.05; **P < 0.01; ***P < 0.001.

The chromosomal locations of the AMRS constituent genes were shown in **Figure 4a**, while their expression profiles in normal versus tumor tissues were depicted in **Figure 4b**. Somatic mutation data were analyzed based on AMRS (**Figure 4c**), with TMB scores calculated for high- and low-risk groups and subsequent Spearman correlation analysis revealing a positive correlation between AMRS scores and TMB (R = 0.1, p = 0.036; **Figure 4d**).

### Clinical correlation and drug sensitivity analysis

Clinical correlation analysis revealed that the patients in the high-risk group presented higher proportions of stage III/IV disease, T3/T4 tumors, N1 lymph node involvement, and M1 distant metastasis, which aligns with the established clinical expectations (**Figure 4e**).

Meanwhile, drug sensitivity analysis identified the top nine statistically significant compounds, among which the low-risk group consistently demonstrated higher ln(IC50) values than the high-risk group across all agents, suggesting enhanced therapeutic sensitivity in high-risk patients (**Figure 4f**).

### Analysis of AMRS and ICI therapy

**Figure 5:**
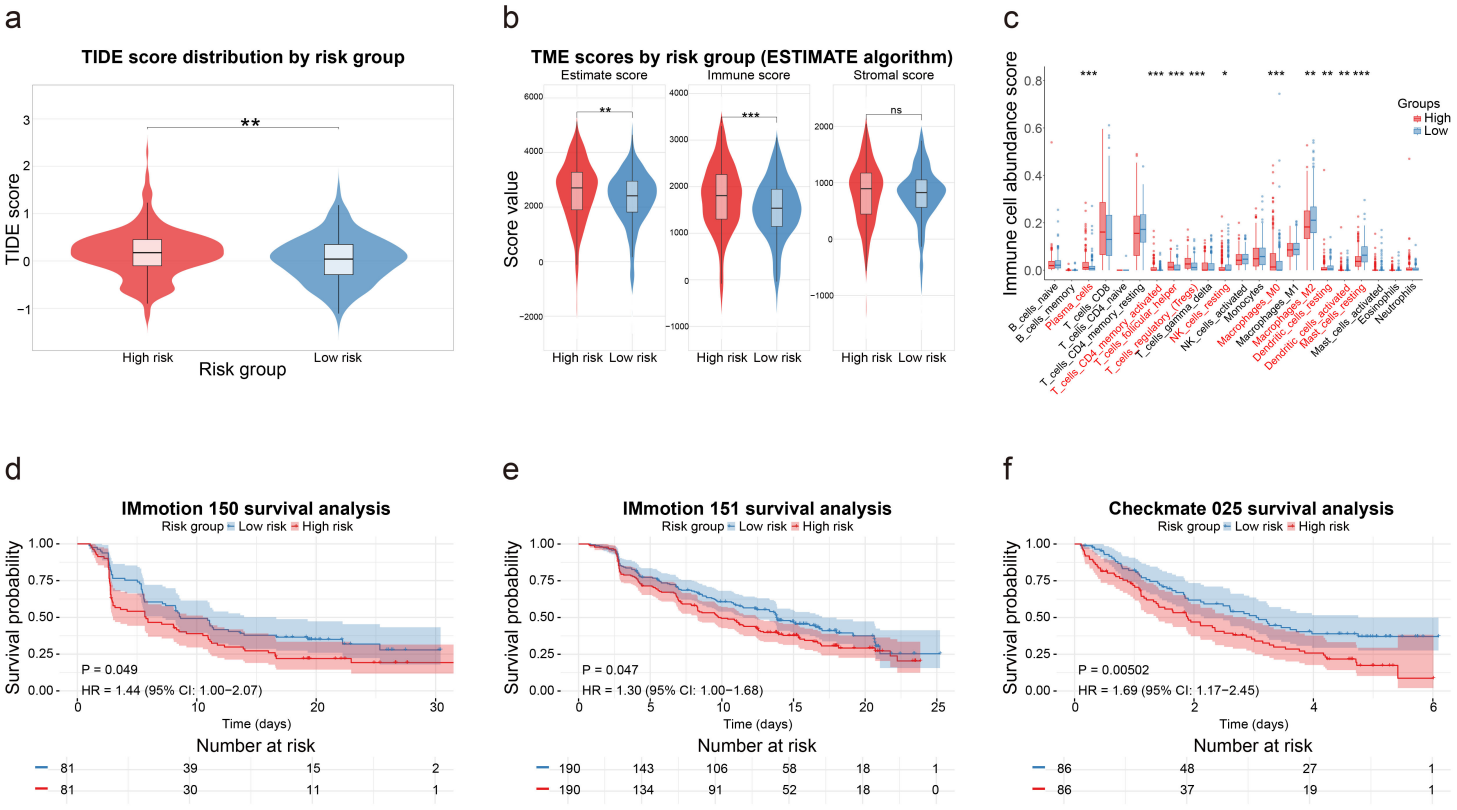
The performance of AMRS in the ICI treatment cohorts. **(a)** The difference in TIDE scores between different risk groups. **(b)** Differences in TME scores analyzed using the ESTIMATE algorithm between different risk groups. **(c)** Differences in immune infiltration analyzed using the CIBERSORT algorithm between different risk groups. **(d–f)** The performance of AMRS in different ICI treatment cohorts in ccRCC. *P < 0.05; **P < 0.01; ***P < 0.001.

In order to further investigate the relationship between AMRS and the TIME in ccRCC, the TIDE, ESTIMATE, and CIBERSORT algorithms were employed to compare the TIME characteristics across risk groups. The analysis revealed significantly higher TIDE scores in the high-risk group (P < 0.05), indicating an increased potential for immune evasion and suggesting possible resistance to ICI therapy, such as anti-PD-1/PD-L1 agents (**Figure 5a**). Concurrently, the elevated ESTIMATE scores reflected a more complex tumor microenvironment in the high-risk group (**Figure 5b**), while CIBERSORT characterized TIME cellular compositions in TCGA (**Figure 5c**). To investigate the associations between AMRS and ICI therapy, survival outcomes were evaluated across risk groups in three ICI cohorts, revealing consistently worse prognoses in high-risk patients (**Figures 5d–f**).

This immunological divergence potentially explains the differential immunotherapy responses between risk groups and confirms the ability of the AMRS to predict ICI treatment outcomes.

### Validation of mRNA expression

To evaluate mRNA expression profiles, we examined the expression levels of four AMGs from the AMRS model in both tumor and adjacent normal tissues. qPCR analysis revealed that CSAD, IL4I1, and P4HA3 were significantly upregulated in tumor specimens, whereas PSAT1 expression was notably reduced in ccRCC tissues (p<0.05, **Supplementary Figure S3a**).

### CSAD functioned as a tumor suppressor in ccRCC

To investigate the functional role of CSAD in ccRCC cells, we first examined its expression across four ccRCC cell lines. qPCR analysis revealed upregulated CSAD expression in all four tumor cell lines, with the highest level observed in 769-P cells and the lowest in OSRC2 cells (**Supplementary Figure S3b**). Consistent with mRNA data, Western blot analysis demonstrated significantly higher CSAD protein expression in multiple tumor samples (T1–T9) compared to their matched normal tissues (N1–N9) (**Supplementary Figure S3c**).

To explore the biological functions of CSAD in ccRCC progression, we knocked down its expression using two specific siRNAs (si-CSAD-1 and si-CSAD-2) in the four cell lines. Knockdown efficiency was confirmed by qPCR (**Supplementary Figure S2**). Colony formation assays showed that CSAD depletion significantly impaired the proliferative capacity of ccRCC cells, with a notable reduction in both the number and size of colonies compared to the negative control (NC) group (**Supplementary Figures S3d, e**).

**Figure 6:**
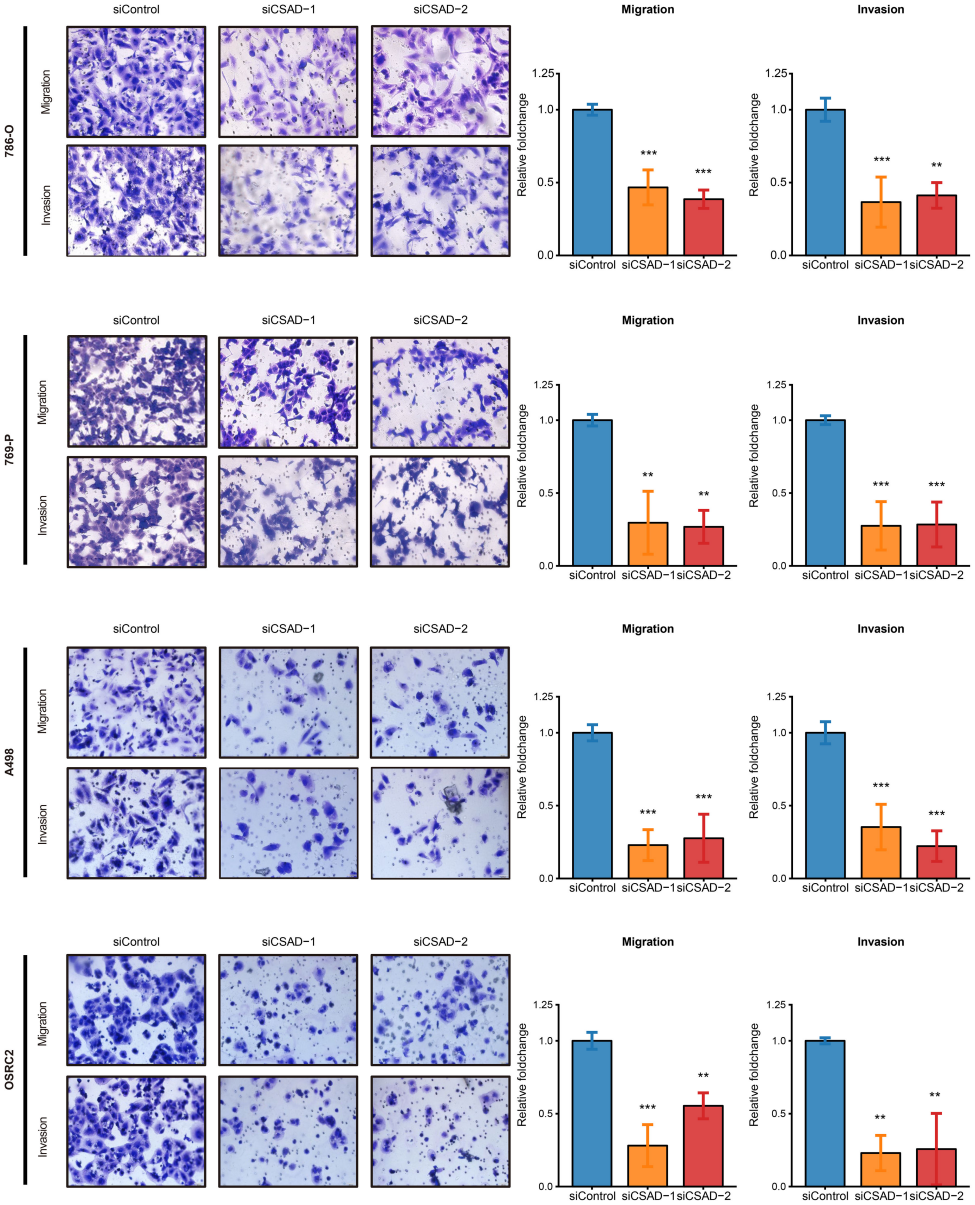
CSAD validation in ccRCC migration and invasion using Transwell assays. *P < 0.05; **P < 0.01; ***P < 0.001.

Furthermore, wound healing and Transwell assays were performed to evaluate the effect of CSAD on cell migration and invasion. Results indicated that CSAD knockdown markedly attenuated the migratory ability of all four ccRCC cell lines. The wound closure rate at 12 hours was significantly lower in si-CSAD groups compared to the NC group, with an average reduction of 25.92% across the four ccRCC cell lines (p < 0.05; **Supplementary Figures S3f, g**). Transwell assays confirmed that CSAD knockdown suppressed both migration and invasion in all cell lines, with migration inhibition generally exceeding 50–70% and invasion inhibition ranging from ~58% to ~78% (**Figure 6**).

In summary, these findings indicate that CSAD is highly expressed in ccRCC and plays a critical role in promoting cancer cell proliferation and migration *in vitro*.

## Discussion

While ICI have become pivotal in renal carcinoma treatment, tumor immune escape occurs through synergistic mechanisms, thereby undermining therapeutic efficacy (31). Current literature confirms that dysregulated ammonia metabolism promotes immune escape by inducing CD8^+^ T cell death (5). However, research on ammonia metabolism in ccRCC remains limited, thus, further investigation into its associations with prognosis and ICI efficacy is crucial to develop novel therapeutic strategies.

Within the TME, ammonia is derived primarily from glutaminolysis: glutaminase catalyzes the conversion of L-glutamine to L-glutamate, releasing ammonia. Conversely, ammonia clearance relies heavily on the urea cycle, which detoxifies ammonia by converting it into the less toxic urea. Glutamine serves as a critical metabolic fuel supporting rapid cellular proliferation and plays essential roles in cancer biosynthesis. In ccRCC, glutamine metabolism is significantly dysregulated, where ammonia generated during its metabolism undergoes reductive carboxylation to form dihydroorotate, thereby, mitigating toxicity while providing nitrogen for nucleotide synthesi (6–8). Additionally, glutamine is competitively uptaken by tumor cells from the TME, depriving CD8^+^ T cells of metabolic substrates and impairing their proliferation and cytokine secretion, concurrently, glutamine depletion activates HIF-1α signaling, prompting macrophages to secrete IL-23, which stimulates regulatory T-cell proliferation and suppresses antitumor immunity (9). Collectively, glutamine metabolic regulators hold significant clinical value in ccRCC.

The urea cycle (UC), which serves as the core metabolic pathway for ammonia detoxification in humans, has its enzymatic expression and impact in tumors remaining debated. In advanced breast cancer, increased argininosuccinate synthetase (ASS1) expression enhances protumor macrophage polarization, suppressing CD8^+^ T-cell activity to facilitate tumor progression and immune escape (32). In contrast, tumors may suppress UC enzymes to redirect carbon/nitrogen resources toward precursor synthesis (e.g., pyrimidines), maximizing biosynthetic efficiency, such as ccRCC exhibits downregulated ASS1/argininosuccinate lyase (ASL), causing ammonia accumulation that drives pyrimidine synthesis (33, 34). Critically, when tumor cells suppress UC enzyme expression, T cells are exposed to a high-ammonia environment, where reactive oxygen species (ROS) disrupt essential amino acid metabolism required for T cells differentiation/proliferation, exacerbating T cells exhaustion and impairing PD-1/PD-L1 therapy responses (35). Additionally, myeloid-derived suppressor cell (MDSC)-secreted ARG1 depletes L-arginine to inhibit T cells function, representing a key immune escape mechanism (36). Given the high immunogenicity and T cells infiltration characteristic of ccRCC, continued investigation into ammonia metabolic pathways (such as glutamine metabolism and the urea cycle) may hold significant implications for improving prognostic outcomes and enhancing ICI therapeutic efficacy (35, 37–39).

The predictive performance of AMRS for ICI efficacy is validated across three independent ccRCC cohorts receiving immunotherapy. Immune infiltration analysis may partially explain this, revealing that high-risk patients are enriched with CD4^+^ activated memory T cells, T follicular helper (Tfh) cells, regulatory T cells (Tregs), and M0 macrophages, while low-risk patients show enrichment of NK cells, dendritic cells, and mast cells. Elevated CD4^+^ activated memory T cells correlate with poorer renal cancer prognosis (40, 41). Critically, Tfh cells and Tregs, which act as key immunomodulators inducing T-cell exhaustion and immunosuppression are significantly increased in the high-risk group, furthermore, Treg infiltration escalates during ccRCC progression, indicating adverse outcomes (42, 43). Additionally, M0 macrophage enrichment in ccRCC creates an adverse microenvironment for PD-L1-expressing CD8^+^ effector T cells, leading to reduced PD-L1 expression and inferior prognosis (44). Conversely, increased proportions of resting dendritic cells and mast cells are associated with improved clinical outcomes (42).

IL4I1, CSAD, P4HA3, and PSAT1 collectively constitute the AMRS, whose biological functions are further explored here. IL4I1, a lysosomal amino acid oxidase, promotes M2 macrophage polarization via the JAK1-STAT3 pathway, inhibiting Tcells function and facilitating ccRCC immune escape and progression (20, 45). P4HA3, which participates in collagen biosynthesis, drives tumor progression in ccRCC and colorectal cancer through the PI3K-AKT-GSK3β axis, conversely, its depletion enhances ICI efficacy in breast cancer (46–48). PSAT1, involved in serine metabolism, is overexpressed in advanced ccRCC and correlates with poor prognosis, its inhibition increases sunitinib sensitivity, particularly in treatment-resistant cases (49, 50). CSAD, modulating taurine metabolism, may be associated with immunotherapy prognosis in liver cancer (43). Although CSAD ranked second in variable importance within the AMRS model (**Figure 3e**), PSAT1 (the highest-ranked gene) has been studied and validated for its prognostic and immunotherapeutic roles in ccRCC (50), whereas the biological functions of CSAD remain poorly characterized. Therefore, CSAD was selected for functional verification *in vitro*. Results demonstrated that CSAD knockdown significantly inhibited ccRCC migration and invasion, suggesting its potential as a novel therapeutic target.

Initially, this study aimed to develop a prognostic model using machine learning algorithms for predicting both clinical outcomes and ICI therapeutic efficacy in patients with ccRCC. However, given the suboptimal performance of the initial model, traditional Cox regression was subsequently employed to develop AMRS, yielding similarly unsatisfactory results. Thus, traditional modeling and machine learning were combined, significantly enhancing predictive performance and demonstrating superiority over standalone methods. Although not unprecedented—Zhang et al. employed a similar methodology to enhance models (51)—we propose that for cancers like ccRCC, characterized by high-dimensional transcriptomic profiles (thousands of genes) coupled with limited clinical samples (hundreds of cases), combining traditional models with machine learning may effectively overcome modeling challenges.

This study has several limitations. First, being a retrospective analysis, it is subject to inherent selection bias that may compromise result accuracy. Second, the predictive capacity of AMRS requires further comparative validation against established models such as the IMDC model. This need is highlighted by a recent study of 657 patients, which reported a C-index of 0.65 for the IMDC model in predicting overall survival in ccRCC—below the common threshold of 0.7 (52). However, the inability to perform a direct comparison due to missing parameters represents a limitation of our study, underscoring the need for further validation of AMRS in external immunotherapy cohorts and prospective multicenter studies. Additionally, more experimental evidence is needed to verify AMGs expression in ccRCC and characterize CSAD functionally.

## Conclusion

This study investigated the role of AMGs in ccRCC, establishing the AMRS that robustly predicts patient prognosis and ICI therapeutic efficacy. Preliminary *in vitro* experiments indicated that CSAD promotes ccRCC cell invasion and metastasis, suggesting its potential as a therapeutic target. For cancers characterized by high-dimensional transcriptomic data yet limited clinical samples, such as ccRCC, integrating traditional models with machine learning may overcome modeling challenges.

## Data Availability

The original contributions presented in the study are included in the article/**Supplementary Material**. Further inquiries can be directed to the corresponding authors.

## Glossary

ccRCC: Clear cell renal cell carcinoma
ICIs: Immune checkpoint inhibitors
IMDC: International Metastatic Renal Cell Carcinoma Database Consortium
LUAD: Lung adenocarcinoma
HCC: Hepatocellular carcinoma
PCD: Programmed cell death
AMGs: Ammonia metabolism-related genes
TCGA: The cancer genome atlas
scRNA-seq: Single-cell RNA sequencing
GEO: Gene expression omnibus
MSigDB: Molecular signatures database
FDR: False discovery rate
KEGG: Kyoto encyclopedia of genes and genomes
GO: Gene ontology
WGCNA: Weighted gene coexpression network analysis
TOM: Topological overlap matrix
PCA: Principal component analysis
t-SNE: t-distributed stochastic neighbor embedding
AMRS: Ammonia metabolism risk score
CNV: Copy number variation
TMB: Tumor mutational burden
TIME: Tumor immune microenvironment
TIDE: Tumor immune dysfunction and exclusion
ATCC: American type culture collection
FBS: Fetal bovine serum
si-NC: small interfering RNA negative control
siCSAD-1: CSAD-targeting small interfering RNA 1
siCSAD-2: CSAD-targeting small interfering RNA 2
RT-qPCR: Real-time reverse transcription quantitative polymerase chain reaction
UC: Urea cycle
ASS1: Argininosuccinate synthetase
ASL: Argininosuccinate lyase
ROS: Reactive oxygen species
MDSC: Myeloid-derived suppressor cell
Tfh: Follicular helper T cell
Tregs: Regulatory T cells.
