# Supplementary material for: A robust ammonia metabolism gene signature identified by machine learning predicts prognosis and immunotherapy response in clear cell renal cell carcinoma
Source: Front Oncol. 2026 Jan 8;15:1709096. doi: 10.3389/fonc.2025.1709096 (PMC12823514; doi:10.3389/fonc.2025.1709096)

| Supplementary Table. S1 Programmed cell death related genes | | | | | | | | | | | | | |
| --- | --- | --- | --- | --- | --- | --- | --- | --- | --- | --- | --- | --- | --- |
| AATF | ABL1 | ACAA2 | ACKR3 | ACVR1 | ACVR1B | ADORA1 | AEN | AGT | AGTR2 | AIFM1 | AKT1 | ANXA6 | APAF1 |
| APPL1 | AR | ARHGEF2 | ARL6IP5 | ARMC10 | ARRB2 | BAK1 | BAX | CASP1 | CASP3 | CASP4 | CASP5 | CASP6 | CASP8 |
| CASP9 | CHMP2A | CHMP2B | CHMP3 | CHMP4A | CHMP4B | CHMP4C | CHMP6 | CHMP7 | CYCS | ELANE | GPX4 | ABCC1 | ACACA |
| ACO1 | ACSF2 | ACSL1 | ACSL3 | ACSL4 | ACSL5 | ACSL6 | AIFM2 | AKR1C1 | AKR1C2 | AKR1C3 | ALOX12 | ALOX15 | ALOX5 |
| ATG5 | ATG7 | ATP5MC3 | BACH1 | ABL2 | ACER2 | ADRA1A | ADRB2 | AMBRA1 | ATF6 | ATG101 | ATG13 | ATG14 | ATG2A |
| ATG2B | ATM | ATP13A2 | ATP6V0A1 | ATP6V0A2 | ATP6V0B | GLUD1 | GLUD2 | FTH1 | PYG | CAPN1 | GLNA | BCL2 | FADD |
| RIPK1 | TNF | TNFRSF1A | TRADD | TRAF2 | PPIA | CAPN2 | HSP90A | IL1A | FDX1 | LIAS | LIPT1 | DLD | DLAT |
| PDHA1 | PDHB | MTF1 | GLS | CDKN2A | GCSH | ATP7A | ATP7B | SLC31A1 | PARP | MIF | HSP70 | PAAN | ARH3 |
| RNF146 | ADPRHL2 | OGG1 | AMPK | BECN1 | CDC42 | CDH1 | CTNNA1 | CYBB | MYH14 | PI3KC3 | RHOA | ROCK | RUBCN |
| UVRAG | MMP1 | MPO | CAMP | PADI4 | EIPA | NCX1 | MIA | ASAH2 | GSDMB | CARS | ATP6V0C | TNFSF6 | ATF3 |
| GSDMC | CBS | ATP6V0D1 | TNFRSF6 | ATF4 | GSDMD | CD44 | ATP6V0D2 | GSDME | CHAC1 | ATP6V0E1 | JNK | ATP2A1 | GZMB |
| CISD1 | ATP6V0E2 | JAK2 | ATP2A3 | HMGB1 | CP | ATP6V1A | CAMK2 | ATP5IF1 | IL18 | CRYAB | ATP6V1B1 | IL1B | AVP |
| CS | ATP6V1B2 | IFNG | BAD | ATP6V1C1 | STAT3 | BAG3 | IRF1 | DPP4 | ATP6V1C2 | IRF9 | BAG5 | IRF2 | EMC2 |
| ATP6V1D | TNFSF10 | BAG6 | NLRC4 | FADS2 | ATP6V1E1 | TNFRSF10A | NLRP1 | FANCD2 | ATP6V1E2 | TNFRSF10B | NLRP2 | FDFT1 | ATP6V1G1 |
| CFLAR | BBC3 | NLRP3 | ATP6V1G2 | XIAP | BCAP31 | NLRP6 | FTL | ATP6V1H | BID | BCL10 | NLRP7 | FTMT | AUP1 |
| NOD1 | G6PD | TRPM7 | BCL2A1 | PLCG1 | GCLC | IFNAR1 | BCL2L1 | PJVK | GCLM | IFNAR2 | BCL2L10 | PRKACA | GLS2 |
| BCL2L11 | IFNGR1 | PYCARD | GOT1 | IFNGR2 | BCL2L12 | SCAF11 | BMF | TLR3 | BCL2L14 | TINAP | GSS | BNIP3 | TIRP |
| BCL2L2 | HMGCR | BNIP3L | IFNA | BCL3 | TP53 | HMOX1 | BOK | IFNB | BCLAF1 | TP63 | HSBP1 | C9orf72 | TRIF |
| BDKRB2 | AIM2 | HSPB1 | CALCOCO2 | VDAC1 | ABCA2 | IKBKB | PGAMS | ABCB9 | NFKB1 | KEAP1 | ACP2 | CA9 | ACP5 |
| CHUK | NRF2 | ADGRE2 | IKBKG | AIRE | AGA | NFKB1A | AP1B1 | RELA | AP1G1 | AP1M1 | AP1M2 | AP1S1 | AP1S2 |
| AP1S3 | AP3B1 | AP3B2 | AP3D1 | AP3M1 | AP3M2 | AP3S1 | AP3S2 | AP4B1 | AP4E1 | AP4M1 | AP4S1 | ARF1 | ARL8B |
| ARSA | ARSB | ARSG | ASAH1 | ATP10B | ATP6AP1 | ATP6V0A4 | BLK | BLOC1S1 | BLOC1S2 | BORCS5 | BORCS6 | BTK | C12orf4 |
| BDNF | GSDMA | IREB2 | CAMKK2 | SLC25A4S | IL6 | PPID | NOD2 | LPCAT3 | CAPNS1 | CYLD | BIK | TIRAP | MAPILC3A |
| RIPK3 | BIRC6 | MAPILC3B | MLKL | MAPILC3C | CDC37 | TRAF5 | MT1G | CDK5 | TLR4 | BMP4 | NCOA4 | CDK5R1 | RBCK1 |
| BMP5 | NFE2L2 | BMPR1B | NFS1 | JAK1 | NOX1 | CISD2 | JAK3 | NQO1 | CLEC16A | TYK2 | CLN3 | STAT1 | BRCA1 |
| OTUB1 | CLU | STAT2 | BRCA2 | PCBP1 | CPTP | STAT4 | BRSK2 | PCBP2 | CSNK2A2 | STAT5A | PEBP1 | CTSA | STAT5B |
| CAAP1 | PGD | CTTN | STAT6 | PHKG2 | DAP | H2A | CASP10 | PRNP | DAPK1 | TNFAIP3 | CASP12 | PROM2 | DAPK2 |
| RNF31 | CASP2 | PTGS2 | DAPK3 | RPL8 | DAPL1 | SAT1 | DCN | VPS24 | SAT2 | DDIT3 | SLC11A2 | DDRGK1 | CASP8AP2 |
| SLC1A5 | DEPDC5 | SLC39A14 | DEPP1 | VPS4 | CAV1 | SLC39A8 | DHRSX | CHMP1 | CCAR2 | SLC3A2 | DNMIL | CHMP5 | CCK |
| SLC40A1 | DRAM1 | SMPD1 | CD14 | SLC7A11 | DRAM2 | CD24 | SOLE | EEF1A1 | CD27 | STEAP3 | EEF1A2 | ZBP1 | CD28 |
| TF | EIF2AK4 | IL33 | CD38 | TFRC | EIF4G1 | CD3E | EIF4G2 | SQSTM1 | VDAC2 | ELAPOR1 | CD5 | VDAC3 | EP300 |
| CD70 | ZEB1 | EPM2A | CD74 | ERCC4 | CDIP1 | ERN1 | BIRC2 | CDKN1A | EXOC1 | BIRC3 | CDKN2D | EXOC4 | EIF2AK2 |
| CEBPB | EXOC7 | PLA2G4 | EXOC8 | FBXL2 | SPATA2 | CHCHD10 | FBXO7 | FAF1 | CHEK2 | FBXW7 | SHARPIN | CIB1 | FEZ1 |
| NOX2 | CBL | CD164 | CD300A | CD63 | CD68 | CD84 | CHGA | CLN5 | CLNK | CLTA | CLTB | CLTC | CLTCL1 |
| CPLX2 | CTNS | CTSB | CTSC | CTSD | CTSE | CTSF | CTSG | CTSH | CTSK | CTSL | CTSO | CTSS | CTSV |
| CTSW | CTSZ | DEF8 | DNASE2 | DNASE2B | ENTPD4 | FAM98A | FER | FES | FGR | FLCN | FOXF1 | FUCA1 | GAA |
| GAB2 | GALC | GALNS | GATA2 | CIDEB | FEZ2 | USP21 | PARP1 | COA8 | FOXK1 | COL2A1 | FOXK2 | CRADD | FOXO1 |
| CREB3 | FOXO3 | CREB3L1 | CRH | CRIP1 | FYCO1 | CSF2 | FZD5 | CSNK2A1 | GAPDH | GATA4 | CTH | GBA | GFAP |
| GNAI3 | GOLGA2 | CUL1 | GPR137 | CUL2 | GPR137B | CUL3 | GPSM1 | CUL4A | GSK3A | CUL5 | GSK3B | CX3CL1 | HAX1 |
| CX3CR1 | HDAC6 | CXCL12 | HERC1 | HGF | CYP1B1 | HIF1A | DAB2IP | DAP3 | HSP90AA1 | HSPA8 | HSPB8 | HTR2B | DAXX |
| HTRA2 | DBH | HTT | DCC | HUWE1 | DDIAS | IFI16 | DDIT4 | DDX3X | IL10 | DDX47 | IL10RA | DDX5 | IL4 |
| DEDD | IRGM | DEDD2 | ITPR1 | DELE1 | KAT5 | DEPTOR | KAT8 | DIABLO | KDM4A | DIDO1 | KDR | DNAJA1 | DNAJC10 |
| KIF25 | GCC2 | GGA1 | GGA2 | GGA3 | GLA | GLB1 | GM2A | GNPTAB | GNPTG | GNS | GUSB | HEXA | HEXB |
| HGS | HGSNAT | HPS6 | HYAL1 | IDS | IDUA | IGF2R | IL13 | IL13RA2 | IL4R | KIF1B | KIT | KXD1 | LAMP1 |
| LAMP2 | LAMP3 | LAMTOR1 | LAPTM4A | LAPTM4B | LAPTM5 | LAT | LAT2 | LGALS9 | LGMN | LIPA | LRRK2 | LYN | M6PR |
| MAN2B1 | MANBA | MAP1LC3A | MAP6 | DNM1L | KLHL22 | DPF2 | KLHL3 | DYRK2 | LACRT | E2F1 | E2F2 | EDA2R | EIF2AK3 |
| ELL3 | LAMTOR2 | ENO1 | LAMTOR3 | LAMTOR4 | EPHA2 | LAMTORS | EPO | LARP1 | ERBB3 | LEP | ERCC6 | LEPR | LGALS8 |
| ERN2 | ERO1A | LRSAM1 | ERP29 | LZTS1 | EYA1 | EYA2 | MAP1LC3B | EYA3 | MAP1LC3C | EYA4 | MAP3K7 | MAPK15 | MAPK3 |
| FAIM | MAPK8 | FAIM2 | MAPT | FAM162A | MCL1 | FAS | MEFV | FASLG | MET | FASTK | MFN2 | FBH1 | MFSD8 |
| MID2 | FEM1B | MIR199A1 | FGA | MIRLET7B | FGB | MLST8 | FGF10 | MT3 | FGFR1 | MTCL1 | FGFR3 | MTDH | FGG |
| MTM1 | FHIT | MTMR3 | FIGNL1 | MTMR4 | FIS1 | MTMR8 | ENIP2 | MTMR9 | FXN | MTOR | FYN | FZD9 | NEDD4 |
| GOS2 | GABARAP | GATAI | NPC1 | MCOLN1 | MILR1 | MRGPRX2 | MYH9 | NAGA | NAGLU | NAGPA | NAPSA | NDEL1 | NEU1 |
| NPC2 | NR4A3 | PDPK1 | PIK3C3 | PIK3CD | PIK3CG | PIP4K2A | PIP4K2B | PIP4P1 | PLA2G15 | PLA2G3 | PLEKHM1 | PLEKHM2 | PPT1 |
| PPT2 | PSAP | PSAPL1 | PTGDR | PTGDS | RAB34 | RAB3A | RAB7A | RAC2 | RUBCNL | S100A13 | SCARB2 | SGSH | SLC11A1 |
| SLC17A5 | SNAP23 | SNAPIN | SNX16 | SNX4 | NPRL2 | GDNF | NRBP2 | GFRAL | NUPR1 | GGCT | OPTN | GHITM | ORMDL3 |
| GNAI2 | OSBPL7 | PAFAH1B2 | GPER1 | PARK7 | GPX1 | PHB2 | GRINA | PHF23 | PIK3C2A | PIK3CA | GSKIP | PIK3CB | GSTP1 |
| PIK3R2 | PIM2 | HDAC1 | PINK1 | HERPUD1 | HIC1 | PIP4K2C | HINT1 | PLEKHF1 | HIP1 | PLK2 | HIP1R | PLK3 | HIPK1 |
| POLDIP2 | HIPK2 | PRKAA1 | HMGB2 | PRKAA2 | PRKAB1 | HNRNPK | PRKAB2 | HRAS | HRK | PRKAG1 | HSPA1A | PRKAG2 | HSPA1B |
| PRKAG3 | PRKD1 | PRKN | HYAL2 | PTPN22 | HYOU1 | ICAM1 | QSOX1 | RAB39B | IFI27 | RAB3GAP1 | IFI27L1 | RAB3GAP2 | IF127L2 |
| IF16 | RAB8A | IFNB1 | RALB | RASIP1 | IGF1 | RBICC1 | IKBKE | RETREG1 | IL12A | RETREG3 | IL19 | RHEB | SORL1 |
| SORT1 | SPAG9 | SPHK2 | STXBP1 | STXBP2 | SUMF1 | SYK | SYTL4 | TCIRG1 | TFEB | TMEM106B | TPP1 | UNC13D | VAMP7 |
| VAMP8 | VPS33A | VPS33B | VPS4A | WASH3P | ZFYVE16 | RIPK2 | RMC1 | IL2 | RNF152 | IL20RA | RNF41 | RNF5 | ROCK1 |
| IL6R | RPTOR | IL7 | RRAGA | INCA1 | RRAGB | ING2 | RRAGC | ING5 | RRAGD | INHBA | INHBB | RUFY4 | INS |
| SCFD1 | ITGA6 | SCOC | ITGAM | SEC22B | ITGAV | SESN1 | ITM2C | SESN2 | SESN3 | ITPRIP | SH3BP4 | IVNS1ABP | SH3GLB1 |
| SIRT1 | JMY | SIRT2 | JUN | SLC38A9 | KDM1A | SMCR8 | KITLG | SMG1 | KRT18 | SNCA | KRT8 | SNRNP70 | LCK |
| SNX32 | LGALS12 | SNX5 | LGALS3 | SNX6 | SOGA1 | LTBR | SOGA3 | LY96 | SPTLC1 | MADD | SPTLC2 | MAEL | MAGEA3 |
| SREBF1 | MAP2K5 | SREBF2 | MAP3K5 | MAPK7 | STBD1 | STING1 | MAPK8IP1 | STK11 | MAPK8IP2 | STUB1 | MAPK9 | SUPT5H | MARCHF7 |
| SVIP | MAZ | SYNPO2 | TAB2 | MDM2 | TAB3 | MELK | TBC1D14 | MFF | TBC1D25 | TBK1 | MIR132 | TEX264 | MIR15A |
| MIR16-1 | TICAM1 | MIR17 | TIGAR | MIR198 | TLK2 | MIR21 | TMEM150A | MIR210 | TMEM150B | MIR221 | TMEM150C | MIR222 | TMEM39A |
| MIR26B | TMEM39B | MIR27B | TMEM59 | MIR449A | TOMM7 | MKNK2 | MLH1 | TP53INP1 | MLLT11 | TP53INP2 | MMP9 | TPCN1 | MNT |
| TPCN2 | MOAP1 | TREM2 | MPV17L | TRIB3 | MSH2 | TRIM13 | MSH6 | TRIM14 | MSX1 | TRIM21 | MUCl | TRIM22 | MUL1 |
| TRIM27 | MYBBP1A | TRIM34 | NACC2 | TRIM38 | NANOS3 | TRIM5 | NBN | TRIM6 | NCK1 | TRIM65 | NCK2 | TRIM68 | NDUFA13 |
| TRIM8 | NDUFS3 | TRIML1 | NFATC4 | TRIML2 | TSC1 | NGF | TSC2 | NGFR | TSPO | NKX3-1 | UBA5 | NLE1 | UBQLN1 |
| NME5 | UBQLN2 | NMT1 | UBQLN4 | NOC2L | UCHL1 | NOG | UFC1 | NOL3 | UFL1 | NONO | UFM1 | NOS3 | ULK1 |
| USP10 | NR4A2 | USP13 | USP30 | OPA1 | USP33 | P2RX4 | USP36 | P2RX7 | P4HB | PAK2 | VPS13C | PAK5 | VPS13D |
| VPS26A | VPS26B | PARP2 | VPS35 | PAWR | WAC | PCGF2 | WASHC1 | PDCD10 | WDFY3 | PDCD5 | WDR24 | PDCD6 | WDR41 |
| PDJA3 | WDR6 | PDK1 | WDR81 | PDK2 | WIPI2 | ZC3HI2A | PDX1 | ZKSCAN3 | PEA15 | ZMPSTE24 | PELI3 | PERP | PF4 |
| PHIP | PHLDA3 | PIAS4 | PIDD1 | PIH1D1 | PIK3R1 | PLAGL2 | PLAUR | PLSCR3 | PMAIP1 | PML | POLB | POU4F1 | POU4F2 |
| PPARD | PPIF | PPM1F | PPP1CA | PPP1R13B | PPP1R15A | PPP2R1B | PPP3CC | PPP3R1 | PRDX2 | PRELID1 | PRKCA | PRKCD | PRKDC |
| PRKRA | PRODH | PSEN1 | PSMD10 | PSME3 | PTEN | PTGIS | PTH | PTPMT1 | PTPN1 | PTPN2 | PTPRC | PTTG1IP | QARS1 |
| RACK1 | RAF1 | RB1 | RB1CC1 | RET | RFFL | RHOT1 | RHOT2 | RNF183 | RNF186 | RNF34 | RPL11 | RPL26 | RPS27L |
| RPS3 | RPS6KB1 | RPS7 | RRP8 | RTKN2 | RTL10 | S100A8 | S100A9 | SCG2 | SCN2A | SCRT2 | SELENOK | SELENOS | SENP1 |
| SEPTIN4 | SERINC3 | SERPINE1 | SFN | SFPQ | SFRP1 | SFRP2 | SGMS1 | SGPL1 | SGPP1 | SH3RF1 | SHH | SHISA5 | SIAH1 |
| SIAH2 | SIVA1 | SKIL | SLC25A5 | SLC35F6 | SLC9A3R1 | SMAD3 | SNAI1 | SNAI2 | SNW1 | SOD1 | SOD2 | SP100 | SRC |
| SRPX | SST | SSTR3 | ST20 | STK24 | STK25 | STK3 | STK4 | STRADB | STX4 | STYXL1 | SYVN1 | TAF9 | TAF9B |
| TCF7L2 | TERT | TFDP1 | TFDP2 | TFPT | TGFB1 | TGFB2 | TGFBR1 | THBS1 | TICAM2 | TIMM50 | TIMP3 | TM2D1 | TMBIM1 |
| TMBIM6 | TMC8 | TMEM102 | TMEM109 | TMEM117 | TMEM14A | TMEM161A | TNFRSF10C | TNFRSF12A | TNFRSF1B | TNFRSF25 | TNFSF12 | TOPORS | TP53BP2 |
| TP73 | TPD52L1 | TPT1 | TRAF1 | TRAF7 | TRAP1 | TRIAP1 | TRIM32 | TRIM39 | TXNDC12 | TYROBP | UACA | UBB | UBE2K |
| UBE4B | UMOD | UNC5B | URI1 | USP28 | USP47 | VNN1 | WDR35 | WNT4 | WWOX | XBP1 | YAP1 | YBX3 | YWHAB |
| YWHAE | YWHAH | YWHAG | YWHAQ | YWHAZ | ZC3HC1 | ZDHHC3 | ZMYND11 | ZNF205 | ZNF385A | ZNF385B | ZNF622 | ZSWIM2 |  |

| Supplementary Table. S2 Ammonia-metabolism related genes | | | | | | | | | | | | | |
| --- | --- | --- | --- | --- | --- | --- | --- | --- | --- | --- | --- | --- | --- |
| CPS1 | ZBP1 | ITGA2 | KCNC1 | SLC7A7 | ABHD12 | BBOX1 | DDO | GCH1 | HDC | MFSD2A | PARK7 | SCLY | TDO2 |
| OTC | CDADC1 | JAK2 | KCNC2 | COX10 | ABHD12B | BCAT1 | DHFR | GCNT4 | HGD | MGST2 | PARS2 | SEPHS1 | TG |
| ASS1 | APOBEC3B | NOS2 | ASIC1 | SLC36A2 | ABHD16A | BCAT2 | DHFR2 | GCSH | HIBADH | MLYCD | PAX8 | SEPHS2 | TGFB2 |
| ASL | GDA | NOS3 | MYD88 | SLC6A19 | ABHD16B | BCKDHA | DHFRP1 | GGH | HIBCH | MMACHC | PCBD1 | SERINC1 | THAP4 |
| ARG1 | CDA | ATP2B4 | NR4A2 | SLC6A18 | ACACA | BCKDHB | DHPS | GGT2P | HMGCL | MMUT | PCBD2 | SERINC3 | THNSL2 |
| NAGS | FTCD | PRODH | OXT | MDH1 | ACACB | BCKDK | DIO1 | GGT3P | HMGCLL1 | MPC2 | PCCA | SERINC5 | TMLHE |
| GLS | SDS | PRODH2 | OXTR | NDUFB10 | ACAD8 | BHMT | DIO2 | GGT5 | HMGCS1 | MPST | PCCB | SHMT1 | TPH1 |
| RHCG | SDSL | ASRGL1 | RRM2B | NDUFC2 | ACADL | BHMT2 | DIO3 | GGT6 | HMGCS2 | MRI1 | PCYOX1 | SHMT2 | TPH2 |
| CAD | HAL | ALDH4A1 | PDE1B | ATP5F1A | ACADM | BLMH | DIP2A | GGT7 | HNF4A | MSRA | PCYOX1L | SLC16A10 | TPO |
| ADSL | CHAC2 | ARHGAP11B | PITX3 | TNFRSF11B | ACADSB | BPHL | DLAT | GGTLC1 | HOGA1 | MTAP | PDHA1 | SLC16A2 | TTC36 |
| ARG2 | PAM | GLYATL1B | PPP3CA | PDP1 | ACAT1 | CARNMT1 | DLD | GGTLC2 | HPD | MTHFD1 | PDHA2 | SLC19A1 | TYMS |
| GLS2 | APMAP | MTHFS | RANBP2 | SLC6A20 | ACCS | CARNS1 | DLST | GGTLC3 | HPDL | MTHFD1L | PDHB | SLC1A2 | TYR |
| RHBG | SRR | ADSS1 | RGS2 | PNPO | ACCSL | CARS1 | DMGDH | GLO1 | HPGDS | MTHFD2 | PDHX | SLC1A3 | UPB1 |
| FH | GGCT | UROC1 | RGS4 | TEFM | ACLY | CARS2 | DPEP1 | GLRX2 | HPN | MTHFD2L | PDK1 | SLC22A4 | VARS1 |
| AGMAT | CHAC1 | AMDHD1 | RGS7 | TARS2 | ACMSD | CBS | DPYD | GLYAT | HSD17B10 | MTHFR | PDK2 | SLC25A12 | VARS2 |
| RHAG | GGACT | CTPS1 | SLC1A1 | COX5A | ACOT12 | CDO1 | DUOX1 | GNMT | HYKK | MTR | PDK3 | SLC25A15 | VNN1 |
| RHD | GATC | SIRT4 | SLC18A2 | AASS | ACSS1 | CGA | DUOX2 | GOT1L1 | IARS1 | MTRR | PDK4 | SLC25A16 | VNN2 |
| RHCE | ASNS | FPGS | TH | SLC7A9 | ACSS2 | CHDH | DUOXA1 | GPT | IARS2 | MVD | PEPD | SLC25A2 | WARS1 |
| ADA | GATB | SLC7A11 | UROS | SLC3A1 | ACY1 | CKB | DUOXA2 | GPT2 | ICMT | MVK | PIPOX | SLC25A21 | WARS2 |
| GNPDA1 | PFAS | PHGDH | PPP1R1B | SLC38A3 | ADI1 | CKM | EARS2 | GPX1 | IDH1 | NARS1 | PLA1A | SLC25A32 | YARS1 |
| RIDA | ASNSD1 | BLOC1S6 | PPP1R9B | SLC25A10 | ADSS2 | CKMT1A | ECHS1 | GSR | IDO1 | NARS2 | PLA2G10 | SLC25A42 | YARS2 |
| ADAR | NADSYN1 | GFPT1 | RGS20 | ATP1B4 | AFMID | CKMT1B | EGLN1 | GSS | IDO2 | NAT8 | PLA2G15 | SLC25A44 | YOD1 |
| ADARB1 | QRSL1 | GGT1 | RGS9 | AQP1 | AGXT | CKMT2 | EGLN2 | GSTA1 | IL4I1 | NAT8L | PLA2G2F | SLC26A7 |  |
| ADARB2 | GMPS | GCLC | HDAC9 | ATP1A1 | AGXT2 | COLQ | EGLN3 | GSTA2 | ILVBL | NDP | PLA2G3 | SLC27A1 |  |
| APOBEC2 | HDAC6 | GCLM | CDK1 | ATP1A2 | AIMP1 | CPQ | ENOPH1 | GSTA3 | INS | NFE2L2 | PLOD1 | SLC39A8 |  |
| ADAT3 | ADAMTS13 | RIMKLA | CLN3 | ATP1A3 | ALDH1A1 | CPT1A | ENOSF1 | GSTA4 | IVD | NFS1 | PLOD2 | SLC45A2 |  |
| ADAD1 | CNR2 | MECP2 | NOXRED1 | ATP1A4 | ALDH1L1 | CPT1B | EPRS1 | GSTA5 | IYD | NOX4 | PLOD3 | SLC46A1 |  |
| GNPDA2 | COMT | AADAT | GLUD1 | ATP1B1 | ALDH1L2 | CPT1C | ETFA | GSTK1 | KARS1 | NUDT7 | PLSCR1 | SLC5A5 |  |
| ADAT2 | ADORA2A | PPAT | GLUD2 | ATP1B2 | ALDH6A1 | CPT2 | ETFB | GSTM1 | KMO | NUDT8 | PM20D1 | SLC5A7 |  |
| APOBEC3D | DBH | CTPS2 | GLUL | ATP1B3 | ALDH7A1 | CRAT | ETHE1 | GSTM2 | KYAT1 | OAZ1 | PM20D2 | SLC6A14 |  |
| LACC1 | NQO1 | NIT2 | PYCR2 | FXYD2 | ALDH8A1 | CROT | ETNK1 | GSTM3 | KYAT3 | OAZ2 | PMVK | SLC6A6 |  |
| ADAL | TRDMT1 | RIMKLB | OAT | PCK1 | ALDH9A1 | CRTAP | FARS2 | GSTM4 | KYNU | OCA2 | PPCS | SLC6A8 |  |
| ADAD2 | DRD1 | TAT | LGSN | PCK2 | AMT | CRYM | FARSA | GSTM5 | LARS1 | ODC1 | PPM1K | SLC7A2 |  |
| DCTD | DRD2 | UCP2 | PYCR1 | SLC9A3 | APIP | CSAD | FARSB | GSTO1 | LARS2 | OPLAH | PRG3 | SLC7A4 |  |
| APOBEC3H | DRD4 | ALDH5A1 | ALDH18A1 | CA2 | ARL6IP5 | CTH | FKRP | GSTO2 | LPCAT3 | OSBPL10 | PSAT1 | SLC7A5 |  |
| APOBEC3A | DRD5 | DGLUCY | PYCR3 | CA4 | ART4 | CTNS | FOLH1 | GSTP1 | LPCAT4 | OSBPL5 | PSPH | SLC7A6 |  |
| APOBEC3F | EDN1 | SLC38A1 | ADHFE1 | SLC4A4 | ASMTL | CTSB | FOLR1 | GSTT1 | LRRC47 | OSBPL8 | PTDSS1 | SLC7A8 |  |
| ADAT1 | EDNRA | ATCAY | DAO | AOC1 | ASPA | CTSK | FOXE1 | GSTT2 | MARS1 | OTUB2 | PTDSS2 | SLCO1C1 |  |
| AMPD1 | FOSB | GLYATL1 | FAH | AOC2 | ASPG | DALRD3 | G6PD | GSTT2B | MARS2 | P4HA1 | PTS | SMS |  |
| AMPD2 | RGS17 | GFPT2 | DDAH1 | AOC3 | ATF4 | DAOA | GAMT | GSTT4 | MAT1A | P4HA2 | QARS1 | SNCAIP |  |
| AMPD3 | GLDC | NR1H4 | GAD1 | AARS1 | ATIC | DARS1 | GARS1 | GSTZ1 | MAT2B | P4HA3 | QDPR | SOD1 |  |
| APOBEC3C | GRIA1 | SLC25A13 | GAD2 | AARS2 | ATP7A | DARS2 | GART | HAAO | MBOAT1 | P4HB | RARS1 | SOD2 |  |
| APOBEC1 | GRIN2A | COX16 | GOT1 | AARSD1 | AUH | DBT | GATA3 | HAGH | MBOAT2 | P4HTM | RARS2 | SULT1B1 |  |
| ADA2 | HDAC2 | LIPT1 | GOT2 | AASDH | AZIN1 | DCT | GATM | HAO1 | MCCC1 | PAH | SARDH | SULT2A1 |  |
| AICDA | NR4A1 | TALDO1 | MIR21 | AASDHPPT | AZIN2 | DDAH2 | GCAT | HARS1 | MCCC2 | PANK2 | SARS1 | TARS1 |  |
| APOBEC3G | HPRT1 | CA5A | NOS1 | ABAT | BAAT | DDC | GCDH | HARS2 | MED1 | PANK4 | SARS2 | TARS3 |  |

Supplementary Fig. S1 The predictive ability of the COX regression model.


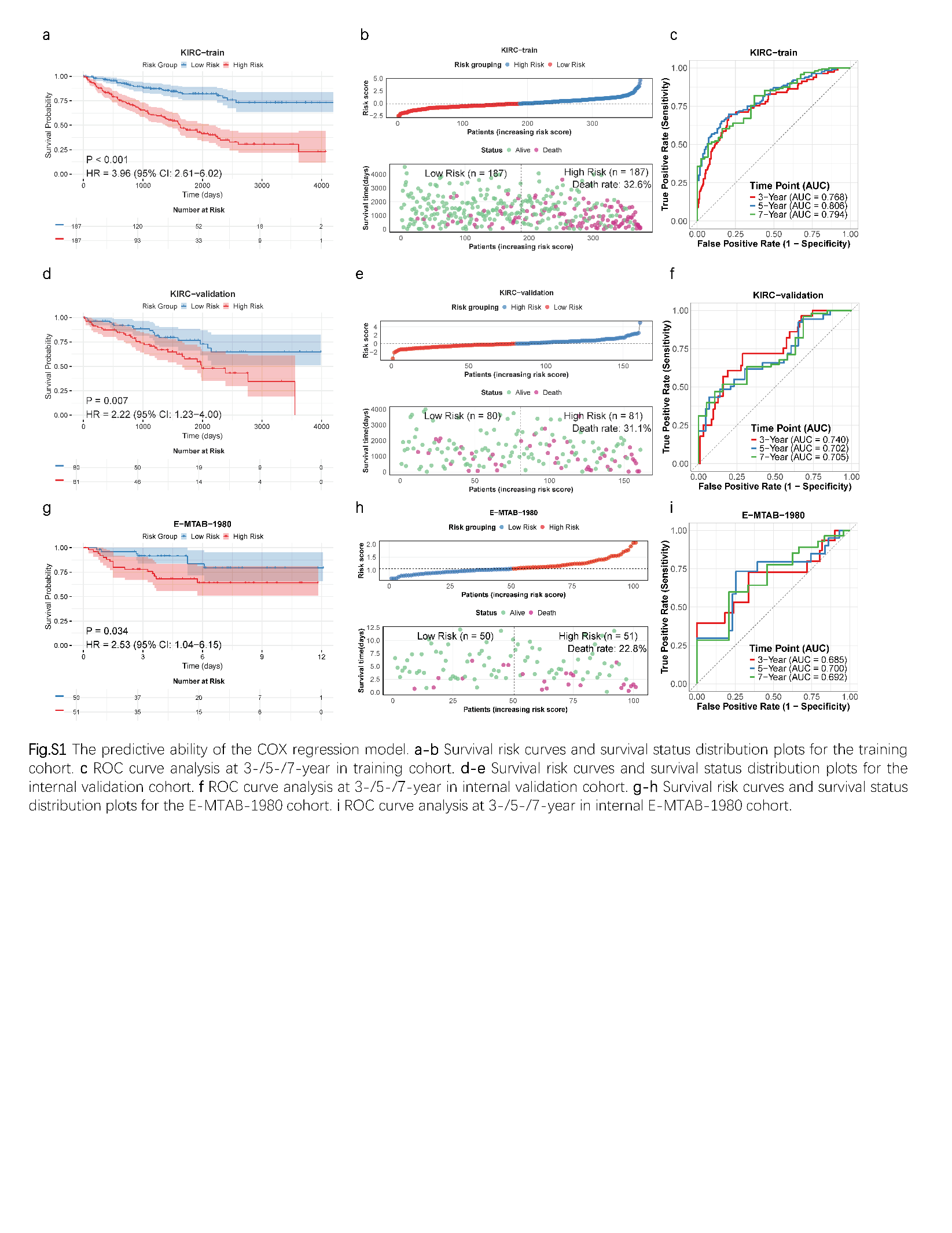


Supplementary Fig. S2 The qRT-PCR assay examined the expression levels of negative control siRNA (si-NC), siCSAD-1, and siCSAD-2 in A498, OSRC2, 786-O, and 769-P cells.
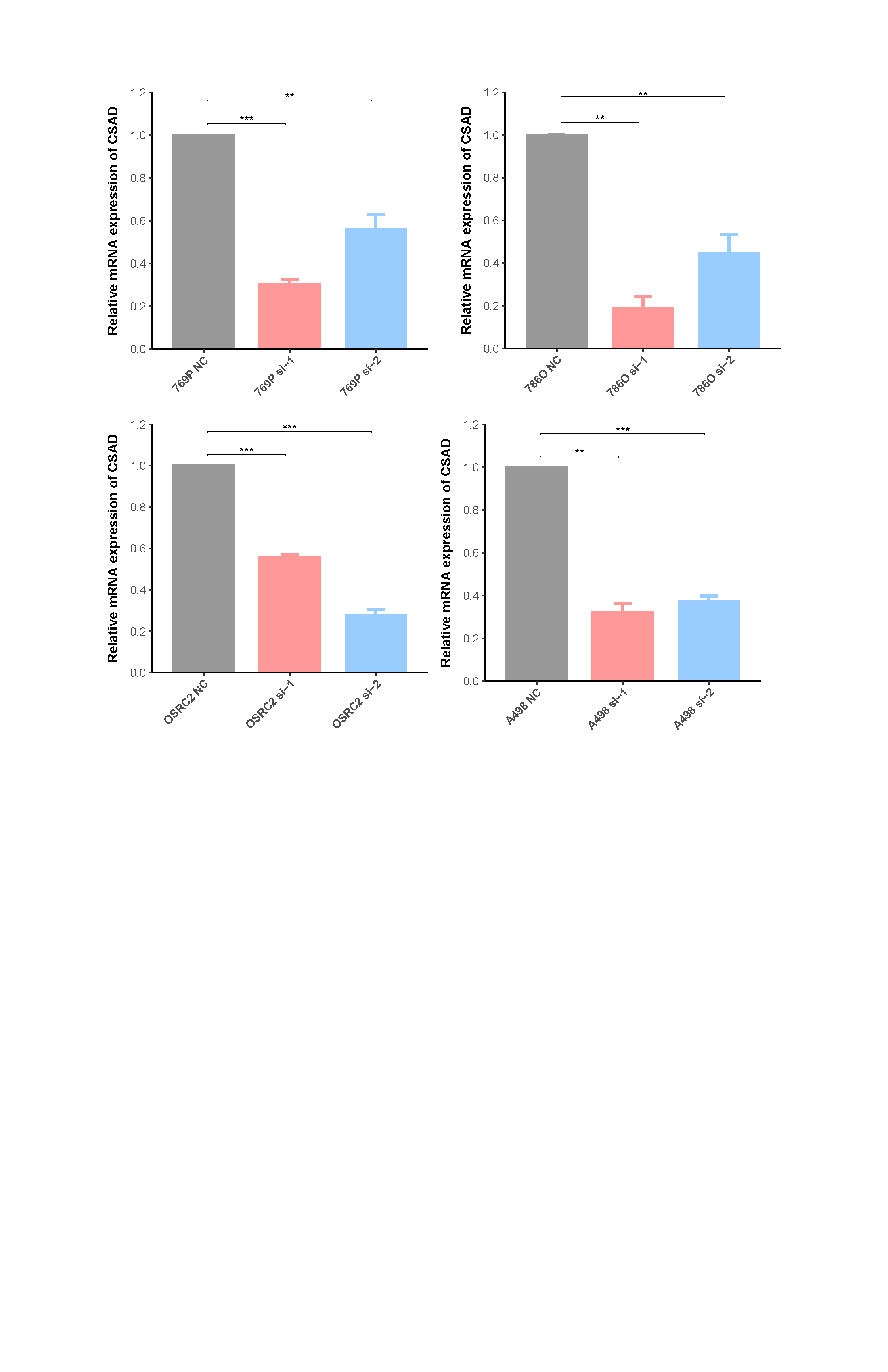


Supplementary Fig. S3 CSAD expression and functional analysis in ccRCC cells in vitro.


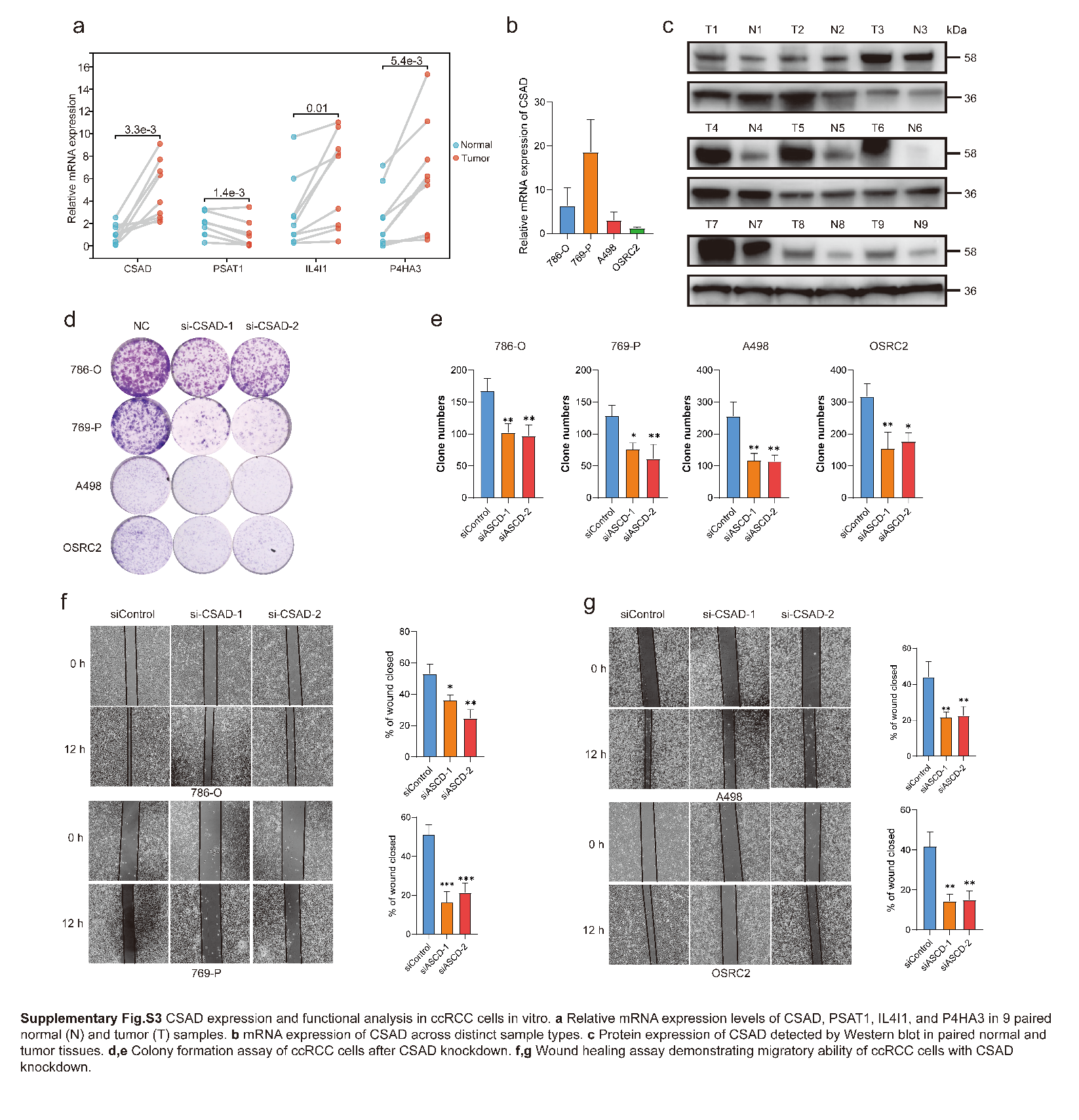

Supplement: Supplementary file 1 [file DataSheet1.docx]
